# Development of a Protein-Rich By-Product by 2^3^ Factorial Design: Characterization of Its Nutritional Value and Sensory Analysis

**DOI:** 10.3390/molecules27248918

**Published:** 2022-12-15

**Authors:** Thamara R. dos Santos, Jakcline dos Santos Melo, Alysson V. dos Santos, Patrícia Severino, Álvaro S. Lima, Eliana B. Souto, Aleksandra Zielińska, Juliana C. Cardoso

**Affiliations:** 1 Department of Pharmacy, Tiradentes University (UNIT), Murilo Dantas Ave. 300, Farolândia, Aracaju 49032-490, SE, Brazil; 2 Post-Graduation Program in Process Engineering, Tiradentes University (UNIT), Murilo Dantas Ave. 300, Farolândia, Aracaju 49032-490, SE, Brazil; 3 Post-Graduation Program in Industrial Biotechnology, Tiradentes University (UNIT), Murilo Dantas Ave. 300, Farolândia, Aracaju 49032-490, SE, Brazil; 4 Technology and Research Institute (ITP), Tiradentes University (UNIT), Murilo Dantas Ave. 300, Farolândia, Aracaju 49032-490, SE, Brazil; 5 Department of Pharmaceutical Technology, Faculty of Pharmacy of University of Porto, Rua Jorge de Viterbo Ferreira, 228, 4050-313 Porto, Portugal; 6 REQUIMTE/UCIBIO, Faculty of Pharmacy of University of Porto, Rua Jorge de Viterbo Ferreira, 228, 4050-313 Porto, Portugal; 7 Institute of Human Genetics, Polish Academy of Sciences, Strzeszyńska 32, 60-479 Poznań, Poland; 8 Post-Graduation Program in Environmental and Health, Tiradentes University (UNIT), Murilo Dantas Ave. 300, Farolândia, Aracaju 49032-490, SE, Brazil

**Keywords:** protein-rich by-product, bee pollen, honey, experimental factorial design, sensory analysis, food technology

## Abstract

The aim of this study was the development of a cereal bar based on bee pollen (BP), honey (H), and flour by-products (peel passion fruit flour—PPFF), generating an innovative product. BP is a protein-rich ingredient and can be used in the composition of cereal bars. PPFF is a by-product rich in fibers. The formulations were developed using a 2^3^ factorial design with four replicates in the center point, studying the sensory analysis as a response variable. The texture and nutritional parameters were performed for the optimal formulation. BP showed ca. 15% of protein. The final formulation (10.35% BP, 6.8% PPFF, and 25% H) presented 22.2% moisture, 1.8% ash, 0.4% total fat, 3.0% fiber, 63.1% carbohydrates, and 74.0 Kcal/25 g. The sensory analysis presented valued around 7 (typical of a traditional bar). Regarding the possibility of purchasing the product, 51% of the panelists said they would probably buy the developed product. The formulated cereal bar had a similar composition as those already marketed. Moreover, it can be considered a source of fiber and is sensory acceptable. This approach opens up new opportunities for developing nutritional and functional foodstuff with improved sensorial aspects.

## 1. Introduction

Snacks based on cereal bars are versatile, nutritious, and healthy products [1]. Several ingredients have been incorporated into cereal bar formulations to generate innovative and competitive products. Other foodstuffs, such as cookies and bread, breakfast cereals, and granola, are also enriched in fibers, proteins, minerals, and vitamins [2,3]. The characteristics of cereal bars, such as nutritional value, production feasibility, pleasant taste, and consumers’ interest in healthy products and diets, are fundamental for their launch into the market and their increased consumption. In the food sector, the association between cereal bars and healthy foods is already quite visible, thereby fostering the search for new healthy alternative products, while responding to circular economy demands [4,5]. Therefore, developing a cereal bar containing bee products, which already have connotations of healthy foods, and passion fruit peel flour with high fiber content may be viable from a nutritional and market point of view [6,7].

Bee products, such as pollen and honey, have nutritional value and functional potential [8,9]. Pollen is a protein source used as a food supplement and consists of carbohydrates, lipids, minerals, and vitamins [10]. Due to its high protein and energy value, it has been used mainly among fans of natural food and athletes as a food supplement. Honey is rich in carbohydrates, primarily glucose and fructose, and contains organic acids, enzymes, vitamins, flavonoids, minerals, and organic compounds [11].

Another product with nutritional and functional potential is passion fruit peel flour, a residue that has high fiber content. Its use in human and animal food has been researched. The results have shown it to be viable as a food source, reducing costs and, at the same time, reducing the problems of eliminating by-products from pulp processing [12]. These products have good nutritional and economic characteristics for formulating cereal bars.

All ingredients and processing of the product are parameters that interfere with the sensory response to nutritional characteristics and the cost of the cereal bar. These parameters can be evaluated by factorial design, which allows determining the formulations to be tested and defining the concentration of the studied variables (honey, pollen, and passion fruit peel flour) necessary for the formulation to influence the sensory response of the product. This work aims to develop a cereal bar formulation based on pollen, honey, and passion fruit peel flour, including its sensorial, nutritional, and physical-chemical characterization as a new foodstuff with market value.

## 2. Results and Discussion

**Figure 1:**
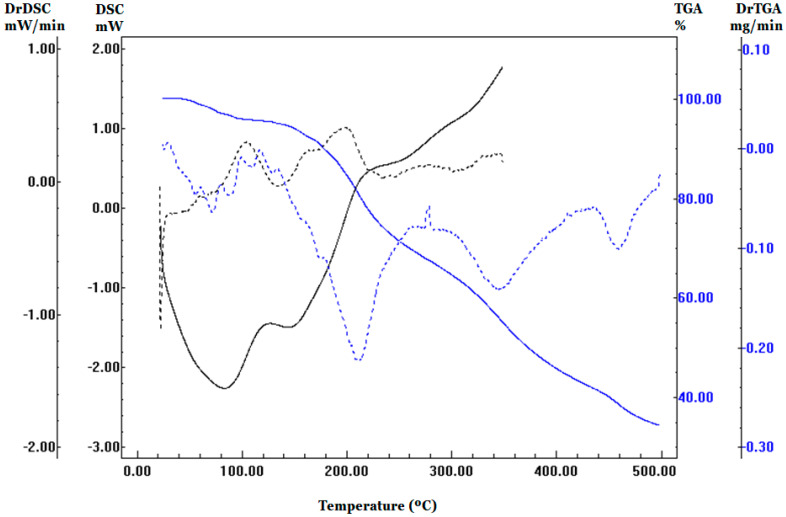
DSC (black profiles) and TGA (blue profiles) analyses of pollen. Dashed lines refer to the second derivates.

The experimental factorial designed implemented for the development of test formulations is shown in Table 1. Figure 1 shows the respective BP derivatives’ calorimetric and thermal decomposition curves. In the thermo-integration profile, it is possible to notice four events (up to 130 °C, between 130 and 275 °C, between 275 and 420 °C, and above 420 °C) well marked on the curve derived from the TGA (blue, dashed line). The first event refers to dehydration with a loss of 4.2% of the mass. Similar profile was observed on DSC curve, showing endothermic events in lower temperature ranges. These results indicate the disorganization of supramolecular structure before thermal degradation.

After amino acid analysis, indirect determination of protein content (15.74% of total amino acids) was obtained, and the results are shown in Table 2. Marchini et al. (2006) [13] reported that pollen collected from Africanized bees *Apis mellifera* L. in Piracicaba (São Paulo region) presented an average of 21.5% crude protein, which respects legislation (minimum of 8%).

**Figure 2:**
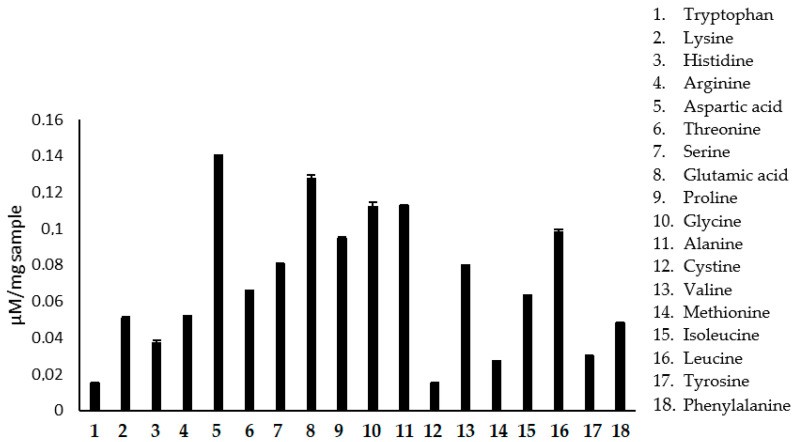
Amino acid composition of pollen.

The increase in the consumption of bee pollen has been observed, mainly among athletes, due to its high protein content and amino acid composition. The interest in knowledge of the amino acids present in the pollen is because it is an essential tool to relate the botanical origin of the product for geographical differentiation of honey. The nutritional value of pollen also depends on its composition of amino acids, as they are a source of proteins and essential amino acids. Another critical point in evaluating the composition of amino acids is the quality control of the product because the amino acids serve as indicators of the freshness and suitability of the pollen drying and storage process, based on the content of a few free amino acids. Our results demonstrate that the pollen presented all essential amino acids and a high concentration of leucine, glutamic acid, and aspartic acid (Figure 2, Table 2). Proline is the free amino acid in greater quantity in both pollen and honey. However, in the results presented in Table 2, it appears that glutamic acid has the highest content in the sample analyzed. This may be due to seasonal and biodiversity variances that may influence the composition of bee products (pollen and honey). Arginine and glutamine are amino acids used by athletes as food supplements. Glutamine is a derivative of glutamic acid, which in the sample studied, showed a high percentage value (1.885%). Supplementation of branched-chain amino acids, such as leucine, isoleucine, and valine, is standard. Leucine is essential in the post-exercise period as it stimulates protein synthesis, accelerating muscle hypertrophy [14]. Valine, isoleucine, and leucine all showed considerable percentage values, suggesting that pollen has potential as a functional food in physical exercise.

Aspartic acid is responsible for the sweet taste of honey. It acts in the Krebs cycle, increasing energy generation, and is also mentioned as an essential amino acid for physical exercise practitioners [15].

Among the foods of animal origin considered sources of proteins are eggs, cow’s milk, and beef. When comparing the protein composition of such foods with pollen, it was observed that the analyzed pollen sample shows higher concentrations of all amino acids, as described in Table 2, except for lysine. The proteic animal source was considered the most relevant, because it provided both quality and quantity of aminoacids. Furthermore, bee pollen protein has its origin in botanical sources. Bees use a great diversity of plant species to acquire pollen components that can provide high-quality protein [16]. BP protein contents compared to vegetal sources also showed improved performance. Pumpkin seed (oil) and soybean meal have around 530 and 460 mg/g, respectively [17].

The results confirm pollen is an essential source of amino acids and can be used generally in the diet. It can also be used in athletes’ supplementation because branched-chain amino acids (isoleucine, leucine, and valine), which provide a significant energy supply for athletes, are in higher concentrations. The results of the composition of the pollen minerals in wet and dry bases are shown in Table 3 [18]. It was observed that pollen could be considered an essential source of minerals (potassium, calcium, magnesium, sodium, and manganese), which are fundamental for maintaining several metabolic functions of the organism [19,20]. The pollen sample showed high concentrations of calcium and magnesium, which require greater daily intake. Milk, considered a calcium-rich food, has about 13,000 µg/g of calcium. Minerals are essential elements for the performance of the body’s metabolic activities. However, depending on their concentration, some may also damage the individual’s health (lead, cobalt, nickel, and cadmium). It was essential to quantify them, and these minerals were found in low concentrations (Table 3) [21].

The pollen showed 26.32% reducing sugar, 1.17% lipids, and 3.24% minerals/ashes. The results indicate pollen is a low-fat, high-energy food through sugars. The pollen had a 12.51 µg/g of pollen equivalent to gallic acid. The content of phenolic acids in the pollen was significant and indicative of the presence of substances with antioxidant power responsible for the sequestration of free radicals in the body. Almaraz-Abarca et al. (2007) evaluated the antioxidant activity of ethanolic extracts of pollen from *P. juliflora*, showing that the substance acted against free radicals and that this biological activity is related to its composition of phenolic acids [22]. It is believed that the intake of exogenous antioxidants may improve the protection of vital cellular components. Such exogenous antioxidants are commonly obtained from food and include vitamins and phenolic compounds. Bee pollen has a critical protein and carbohydrate content, vitamins, and minerals and is a potential polyphenols source, and it is considered a multifunctional food [20]. The phenolic compounds found in bee pollen include flavonoids (kaempferol and quercetin) and phenolic acids (chlorogenic acid), which are well-described molecules in literature as potent antioxidant and anti-inflammatory agents [20].

The instrumental texture analysis has shown that in the concentration range of the ingredients, namely, honey, passion fruit peel flour, and pollen, there was no interference in the results of toughness, hardness, and crispness (*p* > 0.05). Bars were produced with both heat treatment (Table 4) and without heat treatment (Table 5).

Texture has been defined as the primary responsibility of the senses to the physical stimulus resulting from contact between food and some parts of the body. In this way, texture analysis is usually conducted by sensory tests. However, the texturometer has been used to assess the products’ crispness, toughness, and hardness and define limits for measuring the quality [23,24,25]. The texture evaluated by instrumental methods can determine the product’s strength under mechanical action; choose flow properties during processing, handling, and storage; and elucidate the material’s rheological behavior when consumed. The formulations with and without heat treatment showed no significant difference (Table 4 and Table 5). However, when choosing to perform sensory analysis, we opted for formulations subjected to heat treatment, as this is an essential factor in reducing the product’s humidity and assisting in its preservation. The texture evaluated by sensory analysis also showed no significant difference between the formulations proposed in the experimental design. However, it was found that the interaction between passion fruit peel flour and honey was close to substantial (*p* = 0.064). This result suggests greater precision in texture analysis using sensory analysis. In addition, the sensory analysis allows for assessing the regional peculiarities of the test population because the scores attributed to the texture parameter may vary in each region, depending on the type of food that that population is exposed to regularly.

In addition to improving the nutritional aspect of the product, the introduction of fibers in the formulation through the addition of passion fruit peel flour can also interfere with its final texture. The fibers act in the formation of gels, retention of water or lipids, and increasing of viscosity, influencing the surface of the product. Thus, ingredients with high fiber content can be used to improve the food’s texture. The fiber content in the passion fruit peel flour was 38.0%, giving a final average concentration of fibers in the formulation range of 4.9 to 5.9%. These concentrations were estimated from the weight calculation of the proximate composition (Table 6). These values were close to those found in most commercial cereal bars used as a reference in this study. The most common fiber source used in cereal bars is oat. In the last decade, efforts have also been made to introduce agroindustrial residues because these are fiber-rich and cheaper, contributing to circular economy [26,27]. Food can be considered a source of fiber when it contains at least 3 g of fiber/100 g of solid product, and those with a minimum of 6 g of fiber/100 g of concrete product are considered a source of high fiber content.

**Figure 3:**
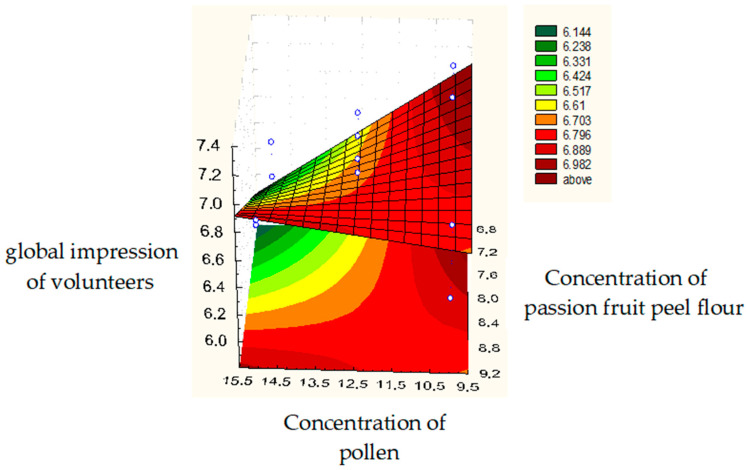
Surface-response chart of volunteers’ global impression of the cereal bar regarding the concentrations of passion fruit peel flour and pollen.

The introduction of passion fruit peel flour, according to our results, also influenced the aroma in the sensory evaluation (*p* = 0.049), but presented values close to the region of significance. In the global impression analysis of the formulations obtained in the experimental design, it was observed that the passion fruit peel flour and pollen concentrations showed a significant influence (*p* = 0.032). Therefore, the optimization region should limit change in the concentrations of all ingredients (Figure 3).

In contrast, honey was added in a relatively high concentration (between 25 and 29%) and had no significant influence on the sensory and instrumental analysis results. This ingredient has remarkable rheological characteristics that can influence its texture and sensory aspect. As the variation studied was small (±2.0%) concerning the central point, it was impossible to detect this ingredient’s influence in the sensory analysis.

Sensory attributes are significant for the acceptance and consumption of the product, and these are evaluated in the following order by the taster: appearance, aroma/fragrance/odor, consistency, texture, and flavor. The set of these attributes generates the global impression of the panelist [28].

Figure 3 shows the influence of pollen concentrations and passion fruit peel flour on the tasters’ perception of the overall impression of the product. It can be observed that the concentration range of these two ingredients with the highest score was between 6.8 to 7.2% for passion fruit peel flour and 9.5 and 10.35% for pollen. From the results, the optimal formulation was defined. This formulation used 10.35% pollen, 6.8% passion fruit peel flour, and 25% honey.

The pollen concentration was set at 10.35% to increase the nutritional contribution in relation to the protein value. In the sensory analysis, the pollen had influence only on the global impression; therefore, it was viable to incorporate it in the highest concentration within the optimized range.

The content of passion fruit peel flour was set at 6.8%, despite the ideal range for incorporating this residue being between this value and 7.2%. The concentration of this ingredient had a significant influence on the aroma and overall impression variable. The increase in its concentration from 6.8% to 7.2% will increase the percentage of fibers in the final product by just 0.15%, which may not increase the purchase appeal when choosing the consumer. An adverse change in aroma and texture can be significant for the consumer’s purchase decision on the product.

The concentration of honey was 25% because, although it does not interfere with the sensory results in the studied range, it can increase the bar’s moisture content. The humidity in honey must be close to 18% to influence viscosity, flavor, crystallization, and conservation. As this ingredient has high moisture content and this variable is directly related to the microbiological quality of the product, the lowest concentration within the studied range was selected for this ingredient for incorporation [29].

Table 6 presents the approximate composition obtained from the optimal formulation and the estimated values for each parameter of the formulations of the experimental design and the commercialized cereal bars (A, B, C, D, E). Despite having a very different flavor and composition, the commercialized bars were selected because they present, for the most part, the presence of honey, or because they are an innovative product (A). The values referring to the commercialized bars (Table 6) were obtained from the labels of the same bars (A, B, E) or the USP Brazilian Food Composition Table (C and D).

Protein content values were found to differ from those theoretically expected; thus, it was necessary to reassess the processing conditions during the analysis verification. However, according to the proximate composition carried out by weight calculation, it is estimated that the cereal bar has a protein content of approximately 10%, which is considered a protein source bar [30]. The protein value of the bar is mainly due to the presence of pollen, an ingredient already mentioned in the literature as a protein-rich product. The protein input of the cereal bar is one of the essential attractions for its commercialization, including for its use by athletes. It was necessary to reassess its concentration in the bar.

It was observed that the cereal bar developed has more than twice the humidity of the others already commercialized, which is a negative factor because the high humidity can facilitate microbial growth and decrease the product’s shelf life. This value may be due to the honey content (25%) used, which is high humidity. Using heat treatment for a more extended period can be an alternative to reduce the moisture content of the bar.

Another factor that can change the moisture content is the loss of volatile substances through processes such as dehydration and volatilization of fructose and other components present in the pollen, which causes a more significant loss of mass and overestimations of the result. The determination of humidity by the AOAC (2000) uses a temperature above 100 °C, which can cause volatilization. Some authors use other methodologies to determine pollen moisture, including a vacuum oven at 70 °C and thermogravimetric analysis (TGA). TGA determines the weight loss of bee pollen as a function of temperature. The first peak appeared until 100 °C and was attributed to the breakdown of hydrogen bonds, resulting in the removal of water and other low-molecule-weight volatile constituents [31].

The ash content of the cereal bar was slightly higher than the others, which was indicative of a higher mineral content that may be due to the pollen concentration. According to an analysis carried out by Funari et al. (2003) [32], pollen had a mineral content of 2.6%, consisting of 0.4% phosphorus, 0.67% potassium, 0.26% calcium, 0.08% magnesium, 0.21% sulfur, 114 ppm iron, 88 ppm zinc, 15 ppm copper, 32 ppm manganese, and 10 ppm boron.

According to the manufacturer’s label, the commercial A, B, and C cereal bars have a lipid content varying between 4 to 8% in their composition. Bars D and E, products considered “light”, have about 3 to 4% of lipids. The lipid content found in the optimized formulation was 0.4% ± 0.06, which was much lower than that found in bars already commercialized. According to Ordinance No. 27/98-SVS/MS, a product can be considered light when it presents a reduction of at least 25% of the content of any of its components. Therefore, the product obtained showed characteristics in its lipid composition classified as “light”.

Carbohydrates, supplied mainly by honey, represent 63.1%, similar to commercial bar B (68.0%). These are mainly responsible for the caloric intake of the cereal bar. However, this contribution is still smaller than that observed in the other bars already commercialized. Thus, this content’s association with the optimized bar’s reduced lipid content is positive, making the product attractive to the consumer.

According to the European Commission nutrition claim guidelines [33], the fiber content should be 3.0% if considering the cereal bar a fiber source. This parameter is crucial, as consumers also evaluate it. The passion fruit peel flour is responsible for this fiber content in this bar because it has a fiber content of 38.0%.

According to the sensory results shown in Table 7, there was no significant difference (*p* > 0.05) between the bars analyzed (optimized and marks A and B) related to appearance. However, concerning the other attributes, the optimized bar and the A bar showed no significant difference between them, which indicates that the developed bar is sensory acceptable with an already commercialized bar, a positive factor for the commercialization of the developed product. Cereal bar A features banana and soy in its composition, the latter of which is an innovative ingredient in a cereal bar. Soy brings a different sensory aspect than that of other bars already on the market. Likewise, pollen and passion fruit peel flour are ingredients that have not yet been explored in this sector and are not part of popularly consumed foods. Because they are innovative products, it is expected that the first contact with the public will present inferior results compared to commercially established products. Cereal bar B, which has banana, oat, and honey in its composition, was used because it has a proximate composition close to that of the developed bars and it is the market leader.

**Figure 4:**
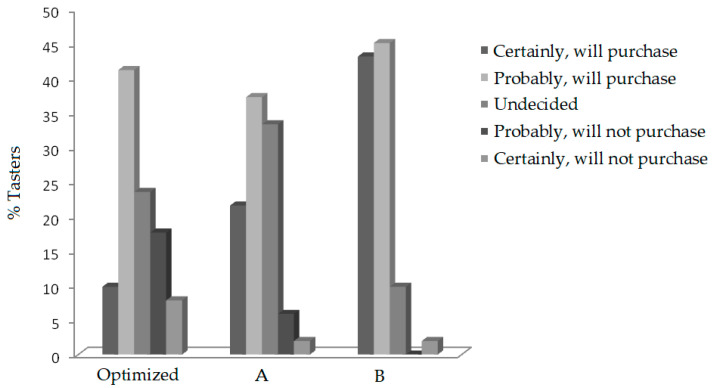
Purchase intention of panelists comparing the optimal cereal bar and the commercialized forms (A and B).

The scores obtained for the optimized bar were close to 7, indicating tasters’ acceptance of the optimized bar, which is a factor favorable to its commercialization that also verified the intentions of the tasters’ purchase (Figure 4).

## 3. Material and Methods

### 3.1. Materials

Passion fruit was obtained from fruits from supermarkets in Aracaju (Sergipe, Brazil) and transported in thermal containers to the Food Research Laboratory of the Institute of Technology and Research (UNIT). The fruits were cut in half, the pulp removed, the peels washed and sanitized (chlorine 50 ppm), and the excess water drained. Commercial bars were also obtained from supermarkets. Crude bee pollen was collected from plants used by *Apis mellifera* L. from locals of the State of Sergipe, Brazil.

### 3.2. Moisture and Thermal Analysis

The moisture content was determined using two methods: dehydration at 105 °C to constant weight and thermogravimetric analysis (TGA). Granulated pollen samples were analyzed in a TA-50WSI Shimadzu (Shimadzu, Kyoto, Japan). The heating rate was 10 °C/min to 120 °C. The percentage of mass loss by the sample was calculated at temperatures of 70 °C, 100 °C, and 105 °C. DSC curves (DSC 2010 TA Instruments, New Castle, DE, USA) were recorded at a temperature range from 25–500 °C, under an N_2_ atmosphere, with a 50 mL/min gas flow rate and a 10 °C/min heating ratio. The aluminium sample holder contained 2 mg of the sample.

### 3.3. Determination of Protein Content

The protein content was determined indirectly by analyzing amino acids. The amino acid analysis was performed in duplicate, and the samples (10–50 mg) were dissolved in 500 µL of HCl (6N), then subjected to vacuum, sealed, and kept at 110 °C for 22 h. After this period, the HCl was evaporated, and the hydrolyzed samples were resuspended in 0.17 M sodium citrate buffer, pH 2.2, containing 400% 15% (*v*/*v*) polyethylene glycol and 0.4% (*v*/*v*) thiodiglycol. The tryptophan in these samples was determined separately after alkaline hydrolysis with 4N lithium hydroxide. All samples were performed in duplicate. Using an automated analyzer, the amino acid analysis of samples hydrolyzed with HCl and LiOH was performed by ion exchange chromatography with post-chromatographic derivation by ninhydrin. A detector at two wavelengths was used: 440 nm for proline and 570 nm for the other amino acids.

### 3.4. Determination of Mineral Composition

The pollen sample (0.2 g) was digested with 5 mL of concentrated nitric acid and ultra-pure water (50 mL). From this solution, the analysis was performed on the Perkin Elmer model AA 300 atomic absorption spectrometer (Bucks, UK) with an air-acetylene flame. The wavelength (λ) used was specific for each mineral researched: Ca 422.7 nm; Cd 228.8 nm; Co 240.7 nm; Cr 357.9 nm; Cu 324.8 nm; Faith 248.3 nm; K 766.5 nm; Mg 285.2 nm; Mn 279.5 nm; Na 589.0 nm; Ni 232.0 nm; and Pb 217.0 nm. For λ less than 300 nm, a deuterium lamp was used for background correction.

### 3.5. Determination of Crude Lipids, Reducing Sugars, and Sulphur Ashes

The bee pollen was also analyzed for the percentages of lipids, reducing sugar, and minerals/ashes, according to the methods recommended by the Association of Official Agricultural Chemists (AOAC, 1975). The percent of crude lipids was determined by chloroform-methanol extraction. The chloroform layer was collected and washed thrice with 100 mL of a NAD (0.1%) solution. The sample was dried, and the weight difference was used to calculate the percentage of crude lipid. The total reducing and non-reducing sugars was determined by modifying AOAC method No. 13.028 for sugars in cereal foods. The quantity of reducing sugar was calculated as maltose for the Maltose-Sucrose Conversion Table (No. 13.030). The nonreducing sugar was reduced after hydrolysis and calculated as sucrose using the same table. Sulphated ashes were determined according to AOAC Method No. 29.014.

### 3.6. Determination of Total Phenolics

With some modifications, pollen samples were evaluated based on the Folin–Ciocalteu colorimetric method [34]. The samples were diluted with distilled water to obtain concentrations of 5, 0.5, and 0.05 mg/mL. To 1.5 mL of each sample, 1.5 mL of 2 N Folin–Ciocalteu reagent and 1.5 mL of 10% sodium carbonate (Na_2_CO_3_) were added. After 1 h of incubation at room temperature, the absorbance was measured in a spectrophotometer at 760 nm, using distilled water as white. In distilled water, gallic acid (2.5 to 12.5 µg/mL) was dissolved and then used to draw the standard concentration curve. The total phenolic values were expressed as gallic acid equivalents (µg of gallic acid/g sample).

### 3.7. Factorial Design

The cereal bar formulation was developed through factorial design 2^3^ with four replications at the central point [35], as depicted in Table 1. This study defined which proportions of the variables studied (honey, pollen, and passion fruit peel flour) interfere with the attributes of the product’s sensory analysis (response variable). Pre-tests were performed to define the levels (−1), (0), and (+1) of the independent variables, considering the technological, economic, and nutritional feasibility. Table 1 shows the factorial design formulations with the concentrations of pollen, passion fruit peel flour, and honey used. Other ingredients were also used in the formulation to impart flavor, assist in agglutination, and obtain the product. The ingredients were gelatin (4.0–5.0%), maltodextrin (2.2–2.7%), rice flakes (15.7–19.3%), and raisin (25.1–30.9%), used in proportional concentrations in all formulations. These adjuvant ingredients are usually added in cereal bars, and the concentration range was selected based on pilot studies to ensure consistent bars.

### 3.8. Production of the Cereal Bars

For the production of the cereal bars, honey and maltodextrin were firstly heated up for 2 min in an oven at 80 °C. After obtaining a homogeneous broth, this was added with pollen, passion peel flour, crushed rice flakes, and raisins. Homogenization of the mixture was carried out under gelatin solution at 60 °C. Molding was performed for a suitable bar shape, which was then transferred to aluminum foil and heated up (180 °C/15 min).

### 3.9. Texture Analysis

The texture analysis was performed through the TAXT Express Enhanced (Stable) texturometer (TAXT Express, Surrey, UK) with a force range of up to 5 kg, using the compression test mode under constant tension in cyclic tests with a knife probe (knife incisor). The parameters used were established according to the equipment manufacturer’s instructions. A pre-test speed of 1 mm/s, initial trigger force of 5 g, test speed of 1 mm/s, test distance of 8 mm, and post-test speed of 10 mm/s were used. The tests were performed at room temperature four days after sample preparation. These were analyzed in triplicate at three points in each sample, determining the area on the curve in g.s (toughness), the initial gradient in g.s^−1^ (crunchiness), and the maximum strength in g (hardness). The significant differences (*p* < 0.05) for each parameter were determined through analysis of variance (ANOVA), using Prism GraphPad software (San Diego, CA, USA) for statistical analysis.

### 3.10. Analysis of Cereal Bar Composition

The composition was determined by analyzing moisture, proteins, ash, crude fiber, lipids, and reducing sugars, as described above. The total carbohydrates were determined by the difference between 100 g of the sample and the total sum of the values of the proteins, lipids, fixed mineral residue, and crude fiber. The total caloric value was calculated by applying the conversion values for the carbohydrates, lipids, and protein. The centesimal composition of the formulations was performed by weight calculation, in which the physicochemical composition of the pollen and honey was used, according to the results of Souza et al. [36]. In contrast, the design of the shell flour passion fruit was carried out according to Santos (2008) [37], while the others according to the information contained on the product labels.

### 3.11. Sensory Analysis

Samples of cereal bars measuring 2 × 2 cm were presented to consumers at room temperature, indicated with three-digit codes in a sensory assessment room with individual booths. The order of presentation was randomly balanced by drawing a table of numbers. The sensory evaluation was carried out by applying a form containing an unstructured hedonic scale of nine points to analyze satisfaction and acceptance. The test was carried out with a team of 30 consumers to explore the 12 formulations of the experimental design and 50 for the analysis of the optimized cereal bar and commercial bars. All participants were consumers of this kind of snack and, thus, considered as potential buyers of this product. The optimized cereal bar was defined from the results obtained by the sensory analysis of the factorial planning formulations and was compared to two commercial cereal bars (A and B). The data obtained were evaluated using the analysis of variance method (ANOVA), with responses with a significant difference being those with *p* < 0.05, computed using GraphPad Prism software (San Diego, CA, USA).

## 4. Conclusions

Our analysis showed that the ingredients did not have any interference with the texture of the product, and the formulations with and without heat treatment did not present significant differences. However, heat treatment is essential to reduce the moisture content and assist in conserving the product. Passion fruit peel flour should be used in lower concentrations within the optimized range, as it significantly influences the bar’s aroma and texture and is an essential factor for product acceptance. The cereal bar developed had a proximate composition close to the bars already commercialized and can be considered a source of fiber. It has a potential for acceptance because the sensory analysis results were immediate to 7 and similar to a cereal bar already commercialized. There is also the possibility of commercialization because, according to the results presented in the purchase intention, approximately 51% of the panelists answered that they would certainly or possibly buy the product.

## Figures and Tables

**Table 1:** Experimental 2^3^ factorial design levels.

Formulation	Honey (%)	Pollen (%)	Passion Peel Four (%)
1	−1 (25)	−1 (10)	−1 (7)
2	+1 (29)	−1 (10)	−1 (7)
3	−1 (25)	+1 (15)	−1 (7)
4	+1 (29)	+1 (15)	−1 (7)
5	−1 (25)	−1 (10)	+1 (9)
6	+1 (29)	−1 (10)	+1 (9)
7	−1 (25)	+1 (15)	+1 (9)
8	+1 (29)	+1 (15)	+1 (9)
9	0 (27)	0 (12.5)	0 (8)
10	0 (27)	0 (12.5)	0 (8)
11	0 (27)	0 (12.5)	0 (8)
12	0 (27)	0 (12.5)	0 (8)

**Table 2:** The concentration of amino acids (mg/g) quantified in: pollen, egg, cow milk, and cow meat.

Amino Acids	Concentration (mg/g Crude Protein)			
	Egg *	Cow Milk *	Cow Meat *	Pollen
Histidine	22	27	34	58
Isoleucine	54	47	48	83
Leucine	86	95	81	130
Lysine	70	78	89	75
Methionine + cystine	57	33	40	59
Phenylalanine + tyrosine	93	102	80	134
Threonine	47	44	46	78
Tryptophan	17	14	12	31
Valine	66	64	50	93
Total	512	504	479	741

* Values obtained from USP Brazilian Food Composition Table.

**Table 3:** Mineral composition of pollen.

Mineral	Wet Base (µg/g)	Dried Base (µg/g)	RDI * (µg/Day)
Cobalt (Co)	<2.0	<2.0	-
Chromium (Cr)	<2.0	<2.0	35
Cooper (Cu)	13.2	15.2	900
Nickel (Ni)	<4.0	<4.0	-
Manganese (Mn)	159.3	183.0	2300
Cadmium (Cd)	2.2	2.5	-
Lead (Pb)	<2.0	<2.0	-
Iron (Fe)	40.7	46.7	8000
Calcium (Ca)	2169.0	2490.0	1,000,000
Magnesium (Mg)	1049.0	1204.0	420,000
Sodium (Na)	252.0	289.0	1,500,000
Potassium (K)	8117.0	9318.0	3,400,000

* RDI—Recommended Daily Intake for adults. (* National Academies of Sciences, Engineering, and Medicine; Health and Medicine Division; Food and Nutrition Board, 2019, [18]).

**Table 4:** Texture analysis of the designed formulations after thermal treatment.

	Area under the Curve (g.s) (Tenacity)	Initial Gradient (g.s^−1^) (Crunchiness)	Maximum Strength (g) (Hardness)
F1	10.50 ± 7.08	433 ± 110	1.97 ± 231
F2	4.34 ± 1.52	579 ± 319	1.41 ± 429
F3	3.25 ± 503	463 ± 106	1.55 ± 265
F4	4.41 ± 1.08	384 ± 121	1.51 ± 537
F5	4.12 ± 1.38	429 ± 44	1.60 ± 213
F6	6.58 ± 1.52	577 ± 31	2.53 ± 191
F7	5.47 ± 1.04	513 ± 159	2.12 ± 520
F8	6.37 ± 1.51	492 ± 66	2.03 ± 180
F9	5.31 ± 1.50	690 ± 173	1.88 ± 1.12
F10	3.13 ± 790	299 ± 99	398 ± 276
F11	3.58 ± 1.97	356 ± 196	1.10 ± 690
F12	3.16 ± 1.62	358 ± 168	971 ± 606

Mean values of nine determinations (3 intra-sample in triplicate) ± standard deviation; F1–F12: Formulations defined by experimental 2^3^ factorial design (Table 1).

**Table 5:** Texture analysis of the designed formulations without thermal treatment.

	Area under the Curve (g.s) (Tenacity)	Initial Gradient (g.s^−1^) (Crunchiness)	Maximum Strength (g) (Hardness)
F1	4.75 ± 8.97	346 ± 252	902 ± 180
F2	4.17 ± 4.98	371 ± 71	972 ± 177
F3	3.33 ± 4.86	464 ± 35	1.35 ± 930
F4	5.75 ± 2.43	491 ± 167	1.96 ± 648
F5	4.88 ± 1.05	445 ± 102	1.82 ± 175
F6	5.59 ± 2.81	393 ± 114	1.92 ± 465
F7	5.66 ± 345	507 ±121	2.12 ± 336
F8	4.92 ± 1.38	653 ± 101	2.15 ± 192
F9	2.99 ± 1.71	452 ± 226	1.39 ± 679
F10	3.67 ± 2.35	277 ± 97	1.10 ± 589
F11	3.85 ± 1.77	223 ± 43	992 ± 528
F12	3.23 ± 1.59	225 ± 75	1.30 ± 455

Mean values of nine determinations (3 intra-sample in triplicate) ± standard deviation. F1–F12: Formulations defined by experimental 2^3^ factorial design (Table 1).

**Table 6:** Physicochemical composition of optimal cereal bars in comparison to commercialized samples.

	A **	B **	C **	D *	E **	F1 a F12 ***	Optimal ***	Optimal
Humidity (%)	NI	NI	NI	9.2	NI	10.9–11.4	11.0	22.2 ± 0.02
Ashes (%)	NI	NI	NI	1.4	NI	1.2–1.4	1.2	1.8 ± 0.01
Total lipids (%)	6.8	8.0	4.8	3.1	4.4	0.8–1.0	0.9	0.4 ± 0.06
Proteins (%)	14.0	6.4	3.2	6.4	4.8	9.2–10.6	10.0	NE
Fibers (%)	19.2	8.4	4.0	6.0	2.4	3.6–4.5	3.7	3.0 ± 0.26
Carbohydrates (%)	48.0	68.0	76.0	79.8	76.0	69.1–70.6	70.6	63.01
Energy (Kcal/25 g)	80.0	91.0	88.0	87.0	90.0	69.4–72.4	72.5	74.0

* Values obtained from USP Brazilian Food Composition Table; ** Values obtained from the labels; *** Results obtained by weight calculation; A, B, C, D, E—Commercial bars; F1–F12: Formulations defined by experimental 2^3^ factorial design (Table 1); NI—No information available; NE—Not evaluated.

**Table 7:** Sensory analysis of optimal cereal bar in comparison to the commercial forms (A and B).

	Optimal	A	B
**Appearance**	6.71 ^a^	6.88 ^a^	7.00 ^a^
**Flavour**	7.14 ^a^	6.88 ^a,b^	6.29 ^b^
**Taste**	6.06 ^a^	6.92 ^a,b^	7.78 ^b^
**Texture**	6.31 ^a^	6.94 ^a,b^	7.59 ^b^
**Global impression**	6.59 ^a^	7.06 ^a,b^	7.63 ^b^

Means with equal letters (a) or (b) in the same line do not differ statistically (*p* > 0.05).

## Data Availability

Not applicable.

## References

[1] Klerks M., Román S., Verkerk R., Sanchez-Siles L. (2022). Are cereal bars significantly healthier and more natural than chocolate bars? A preliminary assessment in the German market. J. Funct. Foods.

[2] Rasane P., Jha A., Sabikhi L., Kumar A., Unnikrishnan V.S. (2015). Nutritional advantages of oats and opportunities for its processing as value added foods—A review. J. Food Sci. Technol..

[3] Curtain F., Grafenauer S. (2019). Comprehensive Nutrition Review of Grain-Based Muesli Bars in Australia: An Audit of Supermarket Products. Foods.

[4] Jamaludin H., Elmaky H.S.E., Sulaiman S. (2022). The future of food waste: Application of circular economy. Energy Nexus.

[5] Aschemann-Witzel J., Stangherlin I.D.C. (2021). Upcycled by-product use in agri-food systems from a consumer perspective: A review of what we know, and what is missing. Technol. Forecast. Soc. Chang..

[6] Durazzo A., Lucarini M., Nazhand A., Souto S.B., Silva A.M., Severino P., Souto E.B., Santini A. (2020). The Nutraceutical Value of Carnitine and Its Use in Dietary Supplements. Molecules.

[7] Durazzo A., Lucarini M., Souto E.B., Cicala C., Caiazzo E., Izzo A.A., Novellino E., Santini A. (2019). Polyphenols: A concise overview on the chemistry, occurrence, and human health. Phytother. Res..

[8] Kostić A.Ž., Milinčić D.D., Barać M.B., Ali Shariati M., Tešić Ž.L., Pešić M.B. (2020). The Application of Pollen as a Functional Food and Feed Ingredient-The Present and Perspectives. Biomolecules.

[9] Pasupuleti V.R., Sammugam L., Ramesh N., Gan S.H. (2017). Honey, Propolis, and Royal Jelly: A Comprehensive Review of Their Biological Actions and Health Benefits. Oxidative Med. Cell. Longev..

[10] Adaškevičiūtė V., Kaškonienė V., Kaškonas P., Barčauskaitė K., Maruška A. (2019). Comparison of Physicochemical Properties of Bee Pollen with Other Bee Products. Biomolecules.

[11] Donkersley P., Rhodes G., Pickup R.W., Jones K.C., Power E.F., Wright G.A., Wilson K. (2017). Nutritional composition of honey bee food stores vary with floral composition. Oecologia.

[12] Weng M., Li Y., Wu L., Zheng H., Lai P., Tang B., Luo X. (2020). Effects of passion fruit peel flour as a dietary fibre resource on biscuit quality. Food Sci. Technol..

[13] Marchini L.C., Reis V.D.A.d., Moreti A.C.d.C.C. (2006). Composição físico-química de amostras de pólen coletado por abelhas Africanizadas *Apis mellifera* (Hymenoptera:Apidae) em Piracicaba, Estado de São Paulo. Ciência Rural..

[14] Mero A. (1999). Leucine supplementation and intensive training. Sport. Med..

[15] Zhao C.J., Schieber A., Gänzle M.G. (2016). Formation of taste-active amino acids, amino acid derivatives and peptides in food fermentations—A review. Food Res. Int..

[16] Diniz M.R., Silva A.G., Carreira L.M.M., de Almeida E.B., Rêgo M.M.C. (2021). Pollen Spectrum of Honey from the Bee Melipona subnitida Ducke (1910) in Restinga in Maranhão State. Floresta Ambiente.

[17] Görgüç A., Gençdağ E., Yılmaz F.M. (2020). Bioactive peptides derived from plant origin by-products: Biological activities and techno-functional utilizations in food developments—A review. Food Res. Int..

[18] Oria M., Harrison M., Stallings V.A., National Academy of Sciences (2019). Dietary Reference Intakes for Sodium and Potassium.

[19] Gaffney-Stomberg E. (2019). The Impact of Trace Minerals on Bone Metabolism. Biol. Trace Elem. Res..

[20] Komosinska-Vassev K., Olczyk P., Kaźmierczak J., Mencner L., Olczyk K. (2015). Bee pollen: Chemical composition and therapeutic application. Evid. Based Complement Altern. Med..

[21] Kashyap R., Ahmad M., Uniyal S.K., Verma K.S. (2019). Dietary consumption of metal(loid)s-contaminated rice grown in croplands around industrial sectors: A human health risk perspective. Int. J. Environ. Sci. Technol..

[22] Almaraz-Abarca N., da Graça Campos M., Ávila-Reyes J.A., Naranjo-Jiménez N., Herrera Corral J., González-Valdez L.S. (2007). Antioxidant activity of polyphenolic extract of monofloral honeybee-collected pollen from mesquite (*Prosopis juliflora*, Leguminosae). J. Food Compos. Anal..

[23] Szczesniak A.S. (2002). Texture is a sensory property. Food Qual. Prefer..

[24] Melati J., Lucchetta L., do Prado N.V., de Oliveira D.F., Tonial I.B. (2021). Physical and sensory characteristics of salty cereal bar with different binding agents. Food Sci. Technol..

[25] Pezzali J.G., Tsai W., Koppel K., Aldrich C.G. (2021). The use of protein binders and sorghum crisps as potential ingredients in a cereal bar for dogs. J. Sens. Stud..

[26] Marques T.R., Corrêa A.D., de Carvalho Alves A.P., Simão A.A., Pinheiro A.C.M., de Oliveira Ramos V. (2015). Cereal bars enriched with antioxidant substances and rich in fiber, prepared with flours of acerola residues. J. Food Sci. Technol..

[27] Rawat N., Darappa I. (2015). Effect of ingredients on rheological, nutritional and quality characteristics of fibre and protein enriched baked energy bars. J. Food Sci. Technol..

[28] Ruiz-Capillas C., Herrero A.M. (2021). Sensory Analysis and Consumer Research in New Product Development. Foods.

[29] Singh I., Singh S. (2018). Honey moisture reduction and its quality. J. Food Sci. Technol..

[30] Commission C.A. (2001). The Codex Guidelines for the Use of Nutrition Claims Adopted by the Codex Alimentarius Commission at its 22nd Session, 1997 and Amended at Its 24th Session. https://www.fao.org/3/y2770e/y2770e07.htm#fn22.

[31] Thakur M., Nanda V. (2020). Exploring the physical, functional, thermal, and textural properties of bee pollen from different botanical origins of India. J. Food Process Eng..

[32] Funari S.R.C., Rocha H.C., Sforcin J.M., Filho H.G., Curi P.R., Gomes-Dierckk S.M.A., Funari A.R.M., Oliveira-Orsi R. (2003). Composição bromatológica e mineral do pólen coletado por Abelhas Africanizadas (*Apis mellifera* L.) na região de Botucatu, no estado de São Paulo. Arch. Lat. Am. Anim. Prod..

[33] Commission E. (2022). https://food.ec.europa.eu/safety/labelling-and-nutrition/nutrition-and-health-claims/nutrition-claims_en#:~:text=A%20claim%20that%20a%20food%20is%20a%20source%20of%20fibre,of%20fibre%20per%20100%20kcal.

[34] Silva A.M., Martins-Gomes C., Souto E.B., Schäfer J., Santos J.A., Bunzel M., Nunes F.M. (2020). *Thymus zygis* subsp. *zygis* an Endemic Portuguese Plant: Phytochemical Profiling, Antioxidant, Anti-Proliferative and Anti-Inflammatory Activities. Antioxidants.

[35] Severino P., Santana M.H., Souto E.B. (2012). Optimizing SLN and NLC by 2(2) full factorial design: Effect of homogenization technique. Mater. Sci. Eng. C Mater. Biol. Appl..

[36] Souza R.C.d.S., Yuyama L.K.O., Aguiar J.P.L., Oliveira F.P.M. (2004). Valor nutricional do mel e pólen de abelhas sem ferrão da região Amazônica. Acta Amaz..

[37] Santos A.V. (2008). Obtenção e Incorporação de Farinha de Casca de Maracujá na Produção de Bolos de Chocolate. Ph.D. Thesis.

